# Mixed Purpuric and Maculopapular Lesions in a Patient with COVID-19: A Case Report

**DOI:** 10.5811/cpcem.2020.6.48617

**Published:** 2020-07-01

**Authors:** Randall Beaupre, Cody Petrie, Alexander Toledo

**Affiliations:** Creighton University Arizona Health Education Alliance, Department of Emergency Medicine, Phoenix, Arizona

**Keywords:** COVID-19, coronavirus, rash, dermatologic

## Abstract

**Introduction:**

The coronavirus disease of 2019 (COVID-19) caused by the novel severe acute respiratory syndrome coronavirus 2 is a global pandemic that expresses itself with a wide variety of presenting symptoms in patients. There is a paucity of literature describing the dermatologic manifestations of the virus, particularly in the United States.

**Case Report:**

Here we present a case of COVID-19 that manifested with a purpuric rash on the lower extremities and a maculopapular eruption on the abdomen in a patient in acute diabetic ketoacidosis and normal platelet count.

**Discussion:**

The reported presenting symptoms of patients with COVID-19 vary greatly. This is the first documented case of COVID-19 presenting with mixed cutaneous manifestations of a purpuric as well as maculopapular rash.

**Conclusion:**

The cutaneous lesions associated with the COVID-19 infection may mimic or appear similar to other well-known conditions. We illustrate a case of COVID-19 infection presenting with purpuric rash on the lower extremities and a maculopapular rash on the abdomen.

## INTRODUCTION

The scant literature to date detailing the dermatologic manifestations of severe acute respiratory syndrome coronavirus 2 (SARS-CoV-2) (COVID-19) describes a presentation that can vary greatly. There have been case reports noting cutaneous lesions such as urticarial,^1^,^2^,^3^ a rash mistaken for dengue fever,^4^ and various plaques.^5^ The most comprehensive description of the cutaneous manifestations of COVID-19 to date has been reported by Galván Casas et al in a nationwide consensus study in Spain. This study describes five basic categories of rashes associated with COVID-19: erythema with vesicles or pustules; other vesicular eruptions; urticarial lesions; maculopapular eruptions; and livedo or necrosis.^6^ In our literature review we found a single case report published in April 2020 that described a patient presenting with COVID-19 and lower extremity purpura. Notably this patient was found to have thrombocytopenia (as low as 66,000 per cubic millimeter) and was diagnosed with concurrent immune thrombocytopenic purpura.^2^ This study, and other case reports, have failed to describe the presence of purpuric lesions in conjunction with a maculopapular rash on the abdomen in a patient with COVID-19.

## CASE REPORT

A 42-year-old Hispanic female with a history of insulin-dependent diabetes mellitus, hypertension, and asthma presented to the emergency department (ED) with four days of worsening abdominal pain, nausea with non-bloody, non-bilious vomiting and two days of rash. Abdominal pain was described as dull and burning in quality, constant in timing with brief periods of intensification, and generalized in location with notably worse pain in the epigastrium and suprapubic regions. The patient reported shortness of breath that she attributed to the abdominal pain. With regard to her rash, she stated that it began on her lower extremities and had spread centrally to her abdomen and upper extremities. It was non-urticarial, painless, and spared the palms, feet and mucosal surfaces. She first noticed the rash approximately 12 hours prior to presentation.

History was also significant for diabetic medication non-compliance, as she stated she had not taken her medications for 2–3 months. Of note, the patient had finished a course of antibiotics the week prior for what she thinks may have been a urinary tract infection. However, further details regarding the antibiotic were unknown. The patient denied diarrhea, fever, chills, cough, and myalgias. She could not identify known sick contacts, and no one else in her family had similar symptoms at that time.

**Image 1:**
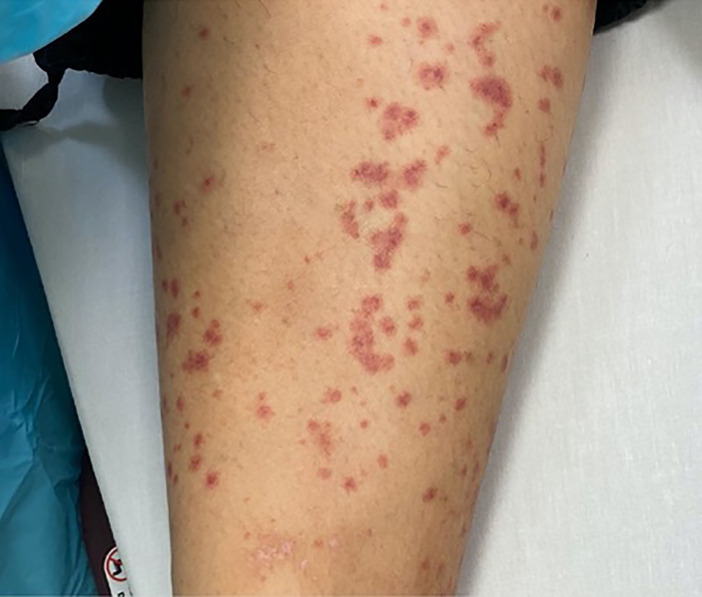
Non-blanching purpuric rash on the bilateral lower extremities of a patient with COVID-19 with concurrent non-blanching maculopapular rash on the abdomen.

**Image 2:**
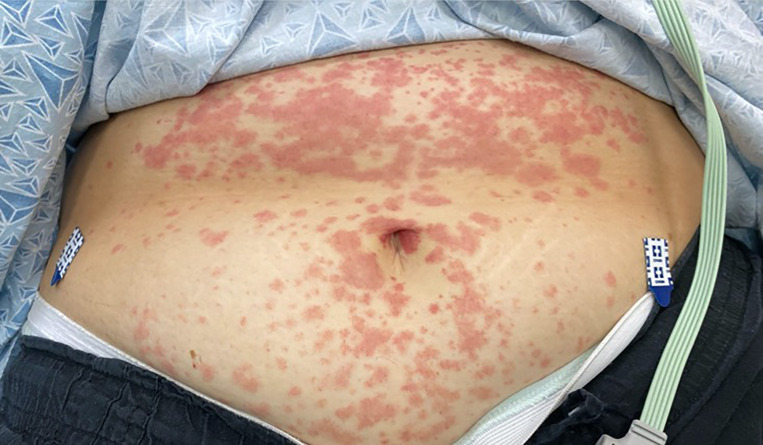
Non-blanching maculopapular rash on the abdomen of a patient with COVID-19 with concurrent non-blanching purpuric rash on the bilateral lower extremities.

On initial evaluation in the ED, the patient’s vital signs were notable for tachycardia to 120 beats per minute, tachypnea to 40 breaths per minute, and blood pressure elevated at 154/103 millimeters of mercury (mmHg). She was afebrile (36.5° Celsius), and oxygen saturation was 93% on room air. She looked ill appearing and was placed in a negative pressure room. Physical examination noted an obese, ill-appearing female who appeared to have Kussmaul respirations. Lungs were clear to auscultation, and cardiac exam was unremarkable. Her abdomen was soft, nondistended, and diffusely tender, most notably in the epigastrium and suprapubic regions but without rebound pain or guarding. Dermatologic examination was notable for a non-blanching purpuric rash on the distal lower extremities (Image 1), as well as a non-blanching maculopapular rash on the abdomen (Image 2).

Initial labs were notable for a platelet count of 660 thousand (K)/microliter (μL) (reference range 157–371 K/μL), prothrombin time of 12.1 seconds (sec) (reference range 11.0–12.5 sec) and an international normalized ratio of 1.1 (reference range <1.1). The patient’s chemistry profile showed a severely decreased bicarbonate level of 7 millimoles (mmol)/liter (L) (reference range 22–26 mmol/L); glucose of 551 milligrams (mg)/ deciliter (dL) (reference range 73–99 mg/dL); and an anion gap of 25 (reference range 3–10).

**CPC-EM Capsule**

What do we already know about this clinical entity?

Five basic categories of rashes are associated with coronaivrus disease of 2019 (COVID-19): erythema with vesicles or pustules; vesicular eruptions; urticarial lesions; maculopapular rash; and necrosis.

What makes this presentation of disease reportable?

In this case, COVID-19 infection presented with purpuric rash on the lower extremities and maculopapular rash on the abdomen.

What is the major learning point?

COVID-19 presentations may vary widely and mimic other diseases. The dermatologic manifestations may be more varied that previously thought.

How might this improve emergency medicine practice?

Early identification of COVID-19 cases based upon known clinical presentations is critical for appropriate patient care as well as public health outcomes.

**Image 3:**
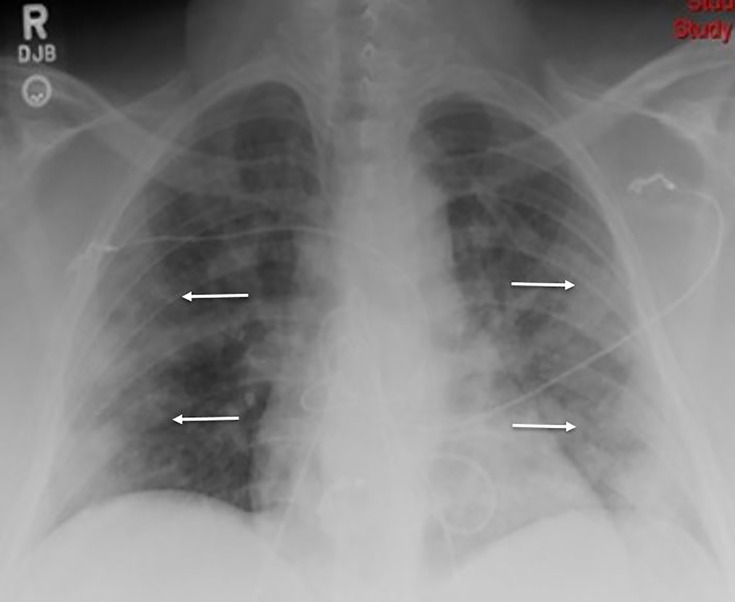
Initial chest radiograph of a patient with COVID-19 showing scattered, bilateral, hazy airspace opacities (indicated by the arrows).

Based upon the patient’s initial vital signs and presentation, we initiated a full sepsis workup. Chest radiograph was performed and demonstrated scattered, bilateral, hazy airspace opacities (Image 3). This was concerning for COVID-19 infection, and so the typical 30 cubic centimeters per kilogram fluid bolus for sepsis was omitted. Initial laboratory values were consistent with diabetic ketoacidosis including a bicarbonate of 7.0 microequivalents/L (mEq/L), elevated anion gap of 25, and a blood glucose of 551mEq/L. Venous blood gas analysis demonstrated that the patient was acidotic with a pH of 7.216 (reference range 7.310 – 7.410). Additionally, her complete blood count revealed a leukocytosis of 13.3 × 10^3^/μL (reference range 4.5 – 11.0 × 10^3^/μL), and an elevated lactic acid of 2.4 millimoles/L (reference range 0.5 – 2.0 mmol/L). Our institutional diabetic ketoacidosis protocol was initiated at this time including an insulin bolus followed by a regular insulin infusion along with maintenance intravenous fluids. The institutional nasopharyngeal COVID-19 rapid test resulted positive.

## DISCUSSION

According to a study done in Wuhan, China, patients infected with COVID-19 occur in a male to female ratio of approximately 1:1 with a median age in the mid-50s.^2^ The most common presenting symptoms of patients with test-confirmed COVID-19 were fever, cough, fatigue, and gastrointestinal symptoms. In this patient population, lymphopenia and eosinophilia were commonly found on laboratory testing. Early data has linked more severe infections with increased number of comorbidities; however, the presence of chronic obstructive pulmonary disease, asthma, and other allergic diseases were not risk factors for contracting the COVID-19 infection.^3^

An article based upon the Italian patient population showed that approximately 20% of patients with COVID-19 infections present with a rash.^1^ This is the first documented case of COVID-19 presenting with mixed cutaneous manifestations of a purpuric as well as maculopapular rash. Further, unlike previous descriptions of patients with COVID-19 and a purpuric rash, this patient did not have thrombocytopenia or idiopathic thrombocytopenic purpura.^2^ Early identification of COVID-19 cases based upon known clinical presentations is critical for appropriate patient care as well as public health outcomes.

## CONCLUSION

The presentation of patients with SARS-CoV-2 varies widely and is just now becoming more understood. The cutaneous lesions associated with this infection may mimic or appear similar to other well-known conditions. We illustrate a case of COVID-19 infection presenting with purpuric rash on the lower extremities and a maculopapular rash on the abdomen.
